# Assessing Residual Gastric Fluid Volume after Administering Diluted Oral Contrast until One Hour Prior to Anesthesia in Children: An Observational Cohort Study

**DOI:** 10.3390/jcm13123584

**Published:** 2024-06-19

**Authors:** Suryakumar Narayanasamy, Robert J. Fleck, Ali I. Kandil, Boma Afonya, Hana Mahmoud, Jiwon Lee, Lili Ding, Mohamed A. Mahmoud

**Affiliations:** 1 Department of Anesthesiology, Cincinnati Children’s Hospital Medical Center, University of Cincinnati College of Medicine, Cincinnati, OH 45229, USA; ali.kandil@cchmc.org (A.I.K.); hana.mahmoud@cchmc.org (H.M.); mohamed.mahmoud@cchmc.org (M.A.M.); 2 Department of Radiology, Cincinnati Children’s Hospital Medical Center, University of Cincinnati College of Medicine, Cincinnati, OH 45229, USA; robert.fleck@cchmc.org; 3 Department of Psychiatry, Icahn School of Medicine at Mount Sinai, 1 Gustave L. Levy Place, New York, NY 10029, USA; boma.afonya@mountsinai.org; 4 Division of Biostatistics and Epidemiology, Cincinnati Children’s Hospital Medical Center, Department of Pediatrics, College of Medicine, University of Cincinnati, Cincinnati, OH 45229, USA; jiwon.lee@cchmc.org (J.L.); lili.ding@cchmc.org (L.D.)

**Keywords:** aspiration, fasting guidelines, NPO, anesthesia, residual gastric volume, sedation, imaging

## Abstract

**Background:** Gastric fluid volume has been used as a surrogate marker for pulmonary aspiration risk in studies evaluating fasting protocol safety. This study measured residual gastric fluid volume in children using a protocol in which diluted oral contrast medium was administered up until one hour before anesthesia. **Methods:** This was a single-center prospective observational cohort trial of 70 children for elective abdominal/pelvic computed tomography (CT). Imaging was performed after diluted enteral contrast medium administration, beginning two hours before and ending at least one hour before induction. For each patient, gastric fluid volume was calculated using an image region of interest. The primary outcome measure was gastric fluid volume measured using the computed tomography image. **Results:** The median time from the end of contrast administration to imaging was 1.5 h (range: 1.1 to 2.2 h). Residual gastric volume, measured using CT was <0.4 mL/Kg in 33%; ≥0.4 mL/Kg in 67%; and ≥1.5 mL/Kg in 44% of patients. Residual gastric volumes measured using CT and aspiration were moderately correlated (Spearman’s correlation coefficient = 0.41, *p* = 0.0003). However, the median residual gastric volume measured using CT (1.17, IQR: 0.22 to 2.38 mL/Kg) was higher than that of aspiration (0.51, IQR: 0 to 1.58 mL/Kg, *p* = 0.0008 on differences in paired measures). Three cases of vomiting were reported. No evidence of pulmonary aspiration was identified. **Conclusions:** Children who receive large quantities of clear fluid up to one hour before anesthesia can have a significant gastric residual volume.

## 1. Introduction

There is tacit agreement that preoperative fasting prior to general anesthesia decreases the risk of pulmonary aspiration. Recent publications have encouraged clear fluid intake until one hour prior to anesthesia induction for elective procedures. Clinical practice guidelines in Europe, Canada, and Australia also endorse this practice [1,2]. However, the American Society of Anesthesiologists’ (ASA) 2023 guidelines continue to recommend clear liquids until two hours prior to general anesthesia in children. They cited insufficient evidence concerning the benefits and harms of liberal fluid intake up to one hour before procedures with general anesthesia [3].

The administration of enteric contrast medium in children who require sedation/anesthesia for abdominal computed tomography (CT) has been a long-standing practice at most large pediatric centers [4]. While a 2010 study of 101 children found that 75% of patients had enteric contrast medium in the stomach 48 ± 5.2 min after the completion of enteric contrast medium ingestion, 50% after 74 ± 7.9 min, and 25% after 135 ± 32.5 min [5], the current goal is to obtain a CT scan within one hour of receiving enteric contrast medium [6] because, in 83% of cases, small bowel transit time is less than two hours. While the average small bowel transit time is one hour and 24 min, transit time can be as fast as 15 min [7]. Waiting several hours after the administration of contrast medium often results in inadequate opacification of the small bowel that can create diagnostic confusion between small bowel loops and fluid collections or masses [8]. Our institution’s anesthetic protocol stipulates that the radiological exam can commence at least one hour after the completion of enteric contrast administration for patients who need sedation or anesthesia after contrast ingestion to minimize the risk of aspiration.

CT can accurately measure increased residual gastric fluid volume [9,10,11], which can put patients at risk for pulmonary aspiration [10,12]. Although there is no strict “volume threshold” over which aspiration risk increases, gastric fluid volumes of up to 1.5 mL/Kg (about 100 mL for an average adult) are often seen in fasted patients [13,14,15,16].

The timing of oral contrast administration before sedation/anesthesia presents a challenge [17,18]. Our institutional fasting guidelines at the time of this study recommended at least two hours of fasting for clear liquid ingestion prior to elective procedures that require anesthesia. Administering enteric contrast medium less than two hours before sedation/anesthesia is at odds with our institutional and ASA pre-procedure fasting guidelines and, in theory, may increase the risk of aspiration [19]. Therefore, this prospective observational study aimed to examine the residual gastric fluid volume when diluted oral contrast is administered up to one hour before sedation/anesthesia for patients who were scheduled for abdominal CT with contrast. We hypothesized that more than 50% of the patients receiving enteric contrast medium within two hours and requiring sedation/anesthesia would have a residual gastric fluid volume of ≥0.4 mL/Kg. This study also aimed to validate the accuracy of measuring residual gastric fluid volume by comparing residual gastric fluid volume measurements via a region of interest in the CT image to using a blind aspiration of the gastric contents with a syringe and a multi-orifice orogastric tube.

## 2. Materials and Methods

This single-center prospective observational cohort trial was conducted within the ethical guidelines outlined in the Declaration of Helsinki and followed Good Clinical Practice. This trial was prospectively registered at clinicaltrials.gov (NCT02239068) https://www.clinicaltrials.gov/study/NCT02239068 (accessed 16 June 2024). The Cincinnati Children’s Hospital Medical Center Institutional Review Board reviewed and approved this study (IRB#2011-0286, PI: Mohamed Mahmoud, initial approval 15 April 2011). Because the study was conducted exclusively in children under the age of ten, written informed consent to participate in the study was obtained from each subject’s legally authorized representative. This report adheres to the Strengthening the Reporting of Observational Studies in Epidemiology (STROBE) guidelines for reporting observational studies [20].

### 2.1. Participants

Children one month to ten years of age (inclusive) scheduled for CT abdomen/pelvis procedures with contrast who required sedation/anesthesia were recruited between June 2011 and April 2021. Patients who violated institutional fasting guidelines before sedation/anesthesia (except for enteric contrast medium) were excluded from the study. Potential subjects were identified from the Radiology CT schedule. During recruitment, all interested potential study participants and/or parents/legal representatives received an oral explanation of the research study. They were told that this study was voluntary, that they could decline participation, and that the information they provided would be kept confidential. In all cases, the research staff explained the study’s purpose and procedures. There was no control group for this study.

### 2.2. Interventions

All patients were sedated/anesthetized using various techniques ranging from rapid sequence induction to sedation with an unsecured airway at the discretion of the anesthesia team.

### 2.3. CT Contrast Protocol

All CT studies were performed with intravenous and enteric contrast medium administration. Studies and reports were reviewed for the documentation of enteric contrast medium administration and the route used for administration (oral, nasogastric tube, gastric tube, or nasojejunal tube). Patients received the enteric contrast medium diluted iohexol 300 mg I/mL (Omnipaque 300, General Electric Healthcare, Princeton, NJ, USA). The contrast was diluted to a final concentration of 6 mg I/mL (1 mL Omnipaque 300/50 mL of water, punch, or juice). The volume of diluted contrast administered was determined by patient age (Table 1). Enteric contrast medium was given orally when possible; if this was not feasible, contrast was administered via nasogastric, gastric, or nasojejunal tubes. The CT gastric image acquisition to determine the residual gastric volume was started at least 60 min after the end of the administration of the oral contrast solution, once the child was sedated/anesthetized.

### 2.4. CT Examinations

All CT examinations were performed following intravenous contrast administration (Ioversol 320 mg I/mL, Optiray 320, Mallinckrodt Inc., Hazelwood, MO, USA). For the CT of the abdomen/pelvis, the volume of intravenous contrast administered was based on body weight (1.5 mL/Kg, 100 mL maximum) (Table 1). The contrast was injected via peripheral intravenous access using a power injector at a rate of 2 mL/second or by hand injection, depending upon the size of the peripheral intravenous access catheter.

When possible, residual gastric fluid volume was aspirated at the conclusion of the CT. This aspiration was performed with a multi-orifice orogastric tube in the supine, right, and left decubitus positions. The total volume of fluid aspirated was measured, and gastric fluid pH was immediately determined using a point-of-care pH test strip kit (Hydrion^®^ test papers and products, Micro Essential Laboratory Inc., Brooklyn, NY, USA).

### 2.5. CT Image Evaluation

**Figure 1:**
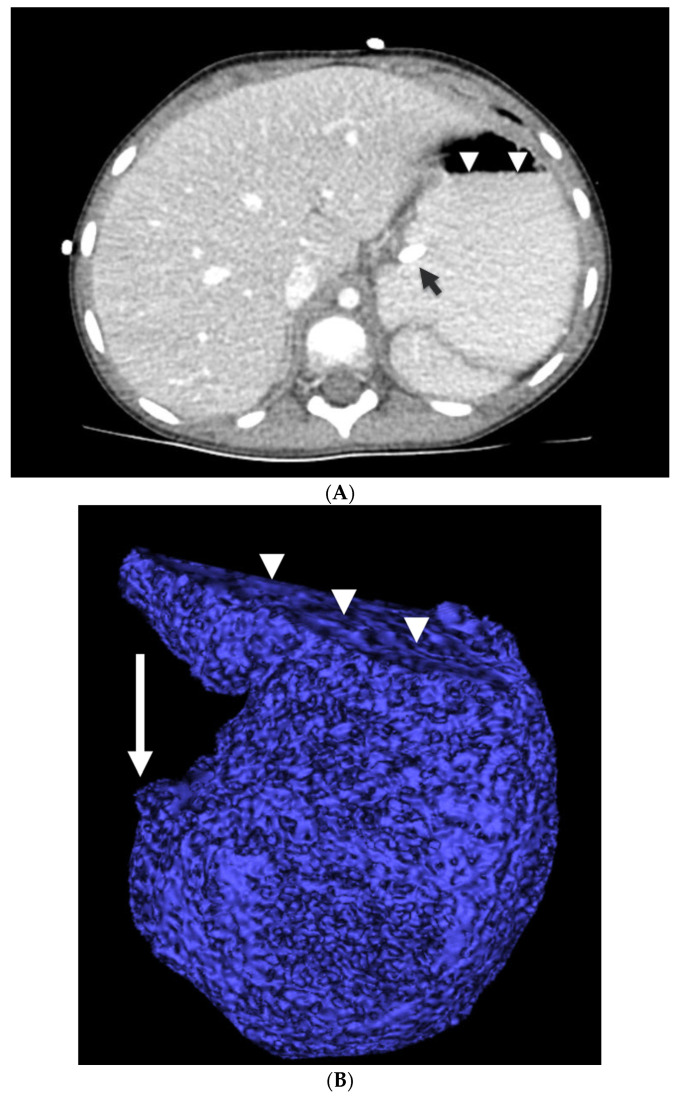
(**A**) Axial 3 mm slice from the CT shows the outline of the dependent fluid filled in the stomach by the arrowheads. The short black arrow points to the enteric tube. Contrast fills the fundus of the stomach and is in contact with the gastroesophageal junction. (**B**) Three-dimensional surface-rendered volume of the stomach contents. Arrowheads indicate the air–fluid interface. The arrow shows the location of the pylorus and the greatly distended antrum proximal to the pylorus. The total volume measures 132 mL. The average density is 168 Hounsfield units, indicating contrast mixed with secretions.

For each patient, the gastric fluid volume was calculated using a region of interest on the CT image. CT images of the abdomen/pelvis were evaluated using Vitrea fX (Vital Images Inc., Minnetonka, MN, USA). Using the sculpt tool in Vitrea, the residual fluid in the stomach was outlined manually with a computer mouse. Regions of the stomach containing high and low attenuation fluid were outlined to obtain a measurement of volume, mean Hounsfield units, and standard deviation. A three-dimensional rendering of the stomach fluid was also produced. (Figure 1A,B).

### 2.6. Outcome Measurements

The primary outcome measure was gastric fluid volume ≥ 0.4 mL/Kg, measured during the CT scan within two hours of sedation via CT calculation using a region of interest. A gastric fluid volume of 0.4 mL/Kg was chosen because, according to several studies, 0.4 mL/Kg was found to be the average gastric fluid volume of fasting pediatric patients [21] Similarly, Wittgrove and colleagues found an average gastric fluid volume of 0.469 mL/Kg in 212 children aged between six months and 21 years who fasted for six hours prior to elective esophagogastroduodenoscopy [22]. Other studies of fasted children found gastric fluid volumes from 0.35 to 0.68 mL/Kg [23,24,25]. Residual gastric fluid volume by aspiration, time from the end of contrast administration to CT scan, gastric pH, airway interventions, and complications related to the administration of oral contrast medium were secondary outcomes. Gastric fluid pH was determined immediately following blind aspiration after the CT scan using a point-of-care test strip. For each patient, the following data were also collected: primary diagnosis; demographic characteristics including age, sex, weight, and ASA physical status; pre- or post-induction intravenous line; agents used for anesthesia/sedation induction and maintenance; airway interventions used (nasal cannula, oral airway, mask, laryngeal mask airway, or endotracheal tube); the method of securing the endotracheal tube (rapid sequence, modified rapid sequence, or none); and complications potentially related to enteric contrast medium administration (e.g., oxygen desaturation, vomiting, coughing, bronchospasm, laryngospasm, or aspiration).

Aspiration was considered to have occurred if any of the following events were documented: aspiration was described in the medical record, chest radiographs were obtained after the procedure to rule out aspiration and showed acute lung opacification, oxygen desaturation or respiratory distress occurred after an episode of vomiting, or the patient required unanticipated admission to the hospital or had other unanticipated changes in therapy related to changes in respiratory status after CT was performed.

### 2.7. Statistical Analysis

All analyses were conducted in SAS 9.4 (SAS Institute, Cary, NC, USA) and R Statistical Software v 4.2.2 (R Core Team, 2021) [26] Statistical significance was determined at *p* ≤ 0.05. Mean and standard deviation or median and interquartile range (IQR) for continuous variables and frequency and percentage for categorical variables were generated for all study variables. All continuous variables were tested for normal distribution. Spearman or Pearson correlation coefficients were derived between continuous variables of interest (for example, between gastric fluid volume from CT and gastric fluid volume from aspiration, and between gastric fluid volume from CT and age, weight, etc.). Two-sample *t*-tests, Wilcoxon rank sum tests, Analysis of Variance, or Kruskal–Wallis tests were used to examine the association between continuous and categorical variables of interest. Differences between paired gastric fluid volume from CT and gastric fluid volume from aspiration were derived and tested for significance using signed-rank tests. The method of limit of agreement was used to derive a 95% limit of agreement, and a Bland–Altman plot was generated.

### 2.8. Power Analysis

Power analysis was based on the primary outcome—the proportion of patients with a gastric fluid volume of ≥0.4 mL/Kg measured during the CT scan within two hours of sedation. A sample size of 70 will have 80% power to detect the difference between the Null hypothesis proportion of 0.5 and an alternative proportion of 0.65 using a one group χ^2^ test with a 5% two-sided significance level.

## 3. Results

**Figure 2:**
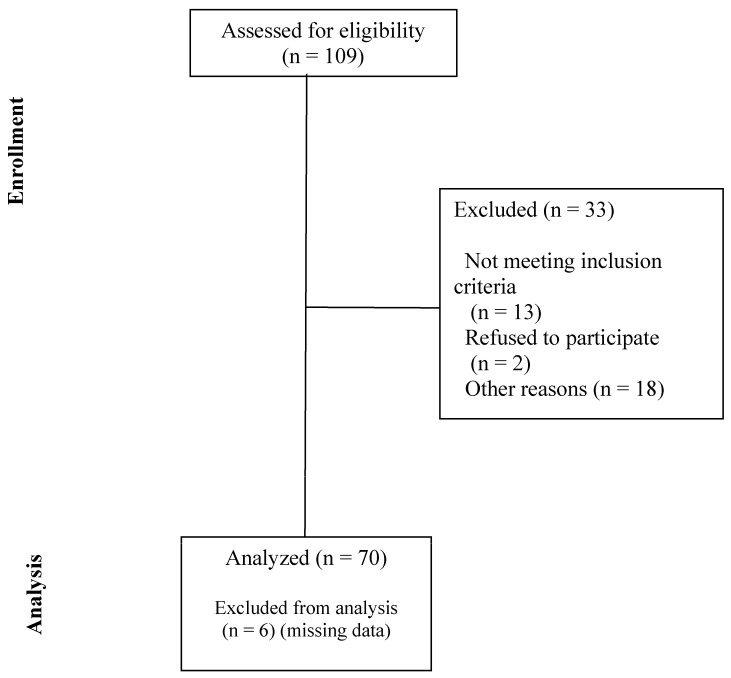
Consolidated Standards of Reporting Trials (CONSORT) diagram.

Between June 2011 and April 2021, 76 children were enrolled in the study (Figure 2). Data from 70 children, ranging in age from 0.3 to 8.1 years, were available for analysis. In total, 46% were female, the median age was 2.8 (IQR 1.6 to 3.9) years, and the median body weight was 13.5 (IQR 11.1 to 15.6) Kg. Detailed descriptive statistics including ASA physical status are shown in Table 2. The range of presenting pathology for patients who received enteric contrast medium and the presenting pathology for 20 patients with the highest gastric fluid volume using CT are shown in Table 3.

### 3.1. Primary Outcome

Gastric fluid volume, as measured during the CT scan within two hours of sedation by CT calculation using a region of interest, was found to be <0.4 mL/Kg in 23 patients (33%) and ≥0.4 mL/Kg in 47 patients (67%). The proportion of children with a gastric fluid volume of ≥0.4 mL/Kg (n = 47, 67% with 95% CI 2-sided (0.548, 0.776), 1-sided (0.567, 1.000)) was significantly higher (2-sided *p* = 0.006, 1-sided *p* = 0.003) than the hypothesized 50%. In total, 67% of patients had a gastric fluid volume of ≥0.4 mL/Kg, 54.2% had one ≥0.8 mL/Kg, and 50% had one ≥1.2 mL/Kg. Importantly, 44.2% of patients had a gastric fluid volume of >1.5 mL/Kg, and 20% had one of >3 mL/Kg. Only six patients had fluid in the fundus and antrum, while the remaining patients had fluid only in the fundus in the supine position.

### 3.2. Secondary Outcomes

#### 3.2.1. Gastric Fluid Volume using CT in Relation to Time

**Figure 3:**
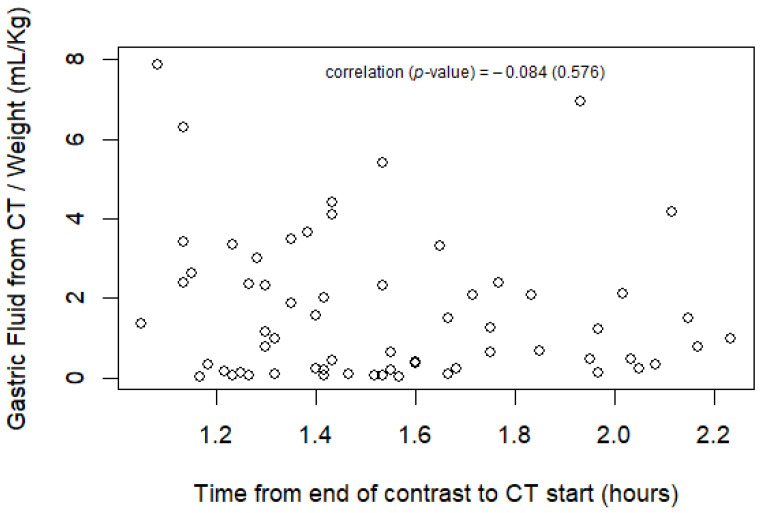
Gastric fluid volume measures using CT in relation to time from end of contrast administration. Scatter plot—time from end of contrast to CT start vs. gastric fluid from CT scan (mL/Kg).

The gastric volumes measured using CT in relation to time from the end of contrast administration are presented in Figure 3. The median time from the end of contrast to CT was 1.5 (IQR 1.3 to 1.8) hours. The range was 1.1 to 2.2 h. The time from the end of the contrast to the start of the CT was not correlated with gastric fluid volume using CT (Spearman’s r = −0.09, *p* = 0.46).

#### 3.2.2. Weight, Age, ASA Status, and Aspiration

**Figure 4:**
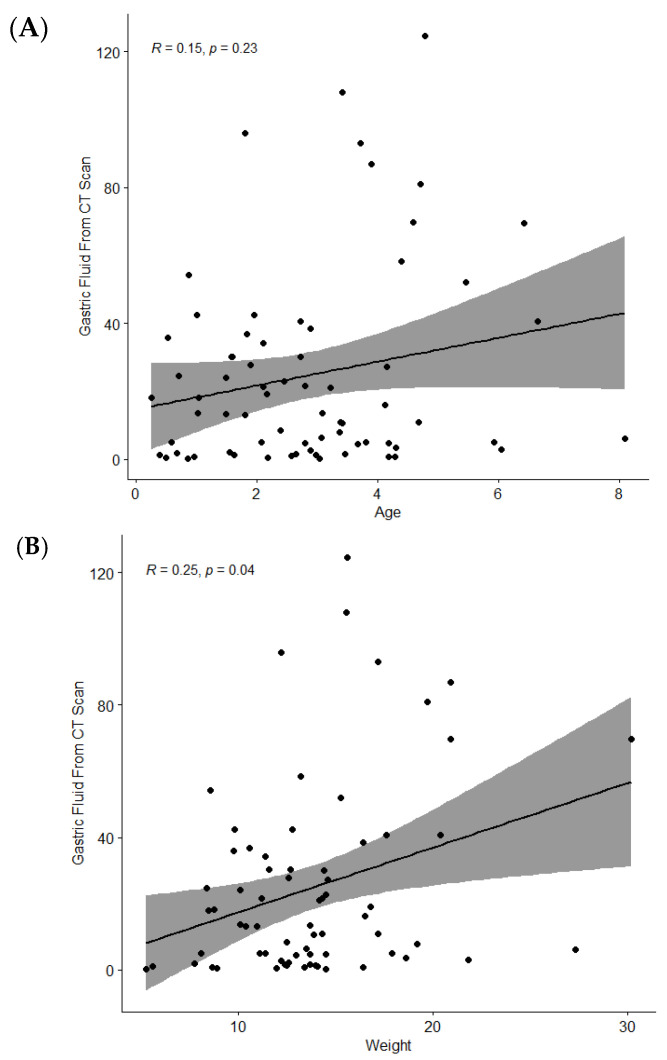
(**A**,**B**): Age and weight correlation with gastric fluid from CT scan.

Gastric fluid volume using CT did not significantly correlate with weight (Spearman’s r = 0.06, *p* = 0.60), age (Spearman’s r = −0.006, *p* = 0.96), sex (*p* = 0.46), or ASA status (*p* = 0.16) (Figure 4A,B). Gastric fluid volume by both CT and aspiration were moderately correlated (Spearman’s r = 0.41, *p* = 0.0003).

#### 3.2.3. Gastric pH

The gastric pH results are presented in Table 2. The time from the end of the contrast administration to the start of the CT was not correlated with pH (Spearman r = −0.08, *p* = 0.58). The gastric pH did not correlate with gastric fluid volume from CT (Spearman r = 0.26, *p* = 0.07), age (Spearman r = −0.27, *p* = 0.06), and weight (Spearman r = −0.27, *p* = 0.06). In 18 patients, the gastric pH could not be measured due to inadequate sample volumes.

#### 3.2.4. Gastric Fluid Volume Measured Using CT vs. Gastric Fluid Volume Measured Using Aspiration

**Figure 5:**
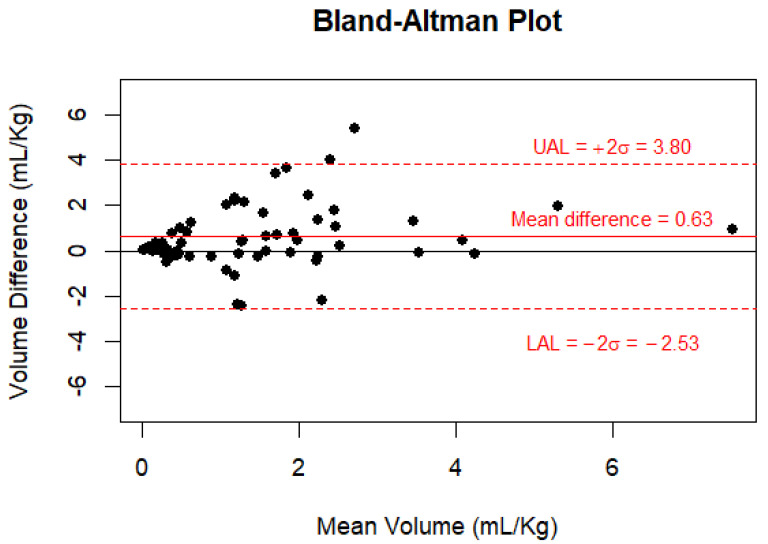
The Bland–Altman plot of gastric fluid volume measured using CT and gastric fluid volume measured using aspiration. The Bland–Altman plot is a graphical method used to assess the agreement or disagreement between two measurement methods. The y-axis shows the difference between the two paired measurements (residual gastric volume/weight from CT − residual gastric volume/weight from aspiration) and the x-axis represents the average of the two paired measurements [(residual gastric volume/weight from CT + residual gastric volume/weight from aspiration)/2]. The black solid reference line corresponds to 0 differences. The red solid line represents the mean difference or overall bias (0.63) between the two measures. The red dotted lines represent the upper (UAL) and lower limits of 95% agreement (LAL), calculated as the mean difference ± 2 × standard deviation of the differences (δ). It is the range within which about 95% of the differences between the two measures are expected to fall. The positive bias (0.63) indicates, on average, that the residual gastric volume/weight from CT is higher than the residual gastric volume/weight from aspiration. This positive bias seems to be due to measurements with a mean volume somewhere between 1 and 4.

Gastric fluid volume was aspirated at the conclusion of the CT scan in 51 patients. Gastric fluid volumes measured using CT and aspiration were moderately correlated (Spearman’s correlation coefficient = 0.41, *p* = 0.0003). The median gastric fluid volume measured using CT was higher (1.17; IQR 0.22 to 2.38 mL/Kg) than the gastric fluid volume measured using aspiration (0.51; IQR 0 to 1.58 mL/Kg). The median of the paired difference between gastric fluid volume using CT and gastric fluid volume using aspiration was 0.13 (IQR 0.10 to 0.97, *p* = 0.0008). Figure 5 shows the Bland–Altman plot of gastric fluid volume measured using CT—gastric fluid volume measured using aspiration. 

#### 3.2.5. Airway Support

Most patients were induced with propofol, and anesthesia was maintained with sevoflurane. The airway support for these patients was as follows: 56 (80%) endotracheal tube, 4 (5.7%) laryngeal mask airway, 1 (1.4%) tracheotomy, 3 (4.2%) spontaneous ventilation via mask, and 6 (8.5%) spontaneous ventilation via nasal cannula. Succinylcholine was utilized during rapid sequence induction for 42 of the 56 patients who underwent endotracheal intubation. There was no evidence of pulmonary aspiration in any patient. Three cases of vomiting occurred; two incidents occurred during extubation and one during induction. Case 1: Three-year-old female with history of recurrent non-bilious vomiting and GERD, vomited a large amount of clear liquids when attempting awake intravenous (IV) line placement prior to rapid sequence induction (RSI) plan, oropharynx suctioned, and airway secured with endotracheal tube with RSI. The lungs were clear to auscultation and no desaturation events were noted. Case 2: A 23-month-old female, admitted after an acute episode of vomiting, general anesthesia with RSI, and endotracheal intubation; the patient vomited at the time of awakening prior to extubation. The endotracheal tube was removed when the patient was fully awake, and no desaturation or aspiration were noted. Case 3: A two-year-old male with two episodes of acute abdominal pain, self-resolving intussusception, and mesenteric lymphadenopathy underwent CT abdomen and pelvis under general anesthesia with RSI and endotracheal intubation. The patient vomited a small amount of emesis (quality of emesis not described); after coughing immediately after extubation, the patient turned to the side and suctioned. The patient was awake and crying with no difficulty in breathing or desaturation episodes.

## 4. Discussion

This single-center prospective observational cohort trial examined the residual gastric fluid volume when a diluted enteric contrast medium is administered up to one hour before sedation/anesthesia in children who were scheduled for abdominal CT with contrast. We found that a significant proportion of patients had a median gastric fluid volume of greater than 0.4 mL/Kg and 1.5 mL/Kg (67% and 44%, respectively). These findings are similar to those of our previous retrospective analysis, where the residual gastric fluid volume exceeded 0.4 mL/Kg in 49% (178/365) of children receiving an abdominal CT who received an oral enteric contrast medium up to one hour before anesthesia/sedation [6].

The median gastric fluid volume, one hour after completing enteric contrast medium, (1.17; IQR 0.22 to 2.38 mL/Kg), was higher than the median gastric fluid volume (0.38; IQR 0.01 to 2.99 mL/Kg) found in our previous retrospective study [6] This difference could be due to the accuracy of the time from the end of contrast administration to capturing the CT image. In our retrospective study, we used data from a chart review, which relies on pre-existing data that can be subject to numerous biases. The current study collected data prospectively, and a research coordinator collected data as per the study protocol, which can reduce sources of bias.

Several studies in children found small preoperative gastric fluid volumes after clear liquids were ingested up to two hours before surgery. A 2012 crossover study in healthy children used magnetic resonance imaging to examine gastric emptying half-life one hour after administration of 3 vs. 7 mL/ Kg of a sugared clear liquid meal after overnight fasting. In patients who received 7 mL/Kg, the median gastric fluid volume was 1.33 (0.30–2.60) mL/Kg, and the gastric emptying half-life was 27 (13 to 43) minutes [27] In 2023, Sarhan and colleagues conducted a randomized double-blinded controlled trial comparing gastric fluid volume in children who fasted for one versus two hours after ingesting a defined volume of clear fluid. Children who received 3 mL/Kg of clear fluid one hour before anesthesia had a median gastric fluid volume of 0.61 (9.41 to 0.9) mL/Kg, more than double the volume after conventional two-hour fasting (0.32; 0.23 to 0.47 mL/Kg). No child in either study group had a gastric fluid volume of ≥1.5 mL/ Kg [28].

The higher frequency of gastric fluid volume ≥1.5 mL/Kg in patients in our study compared to previous studies that used imaging studies or the aspiration of fluid via a gastric tube may be due to two possible factors. The first and most likely is that patients in our study received a larger volume of fluid per kilogram (minimum 10 mL/Kg). The second is that comorbidities in our study populations may have affected gastric emptying. Although none of our patients had documented gastrointestinal functional disturbance, comorbidities may have indirectly affected gastric emptying. Infection, diabetes, hypothyroidism, neurologic disorders, and metabolic disorders are a few of the risk factors for delayed gastric emptying. The patient populations in most studies that examined gastric fluid volume were ASA I or II, where it may be possible for these children’s well-being to allow for gastric emptying to baseline values after one hour [28,29,30]. Only 25% of our study participants were ASA class I/II. Though our results did not show any correlation between ASA status and residual gastric fluid volume, we believe that coexisting diseases may have contributed to the higher gastric residual volumes found in our study. While the composition, osmolarity, and sugar and protein content of the undiluted contrast medium are different than a routine preoperative clear liquid such as water or apple juice, it was very diluted (1 mL contrast in 50 mL of water, apple juice, or punch) and likely mimics physical characteristics of a preoperative clear liquid drink.

Our approach to measuring the gastric fluid volume via CT imaging, which is a standard and accepted technique for volume measurement in radiology [9,10] and bariatric surgery [11], was different from the methods used in previous studies, which measured gastric fluid volumes by using blind aspiration of the gastric contents [21,22], ultrasound assessment [28,29,31], or magnetic resonance imaging [27]. This was a practical choice because all our study patients were undergoing abdominal CT scans. As per previous studies, we measured gastric fluid volume using blind aspiration through multi-orifice catheters from the supine, left, and right decubitus positions [21,32]. With this method, 96–97% of gastric fluid volume is recovered [21,33], whereas only 53 or 78% was recovered in other studies [34,35]. Since blind aspiration may underestimate gastric fluid volume, an expected finding of our study was the suboptimal accuracy of measuring gastric fluid volume via the blind aspiration method. In our study, the median gastric fluid volume measured using CT was higher (1.17; IQR 0.22, 2.38 mL/Kg) than the gastric fluid volume measured using aspiration (0.51 mL/Kg; IQR 0, 1.58 mL/Kg), which confirmed our expectations.

A recent study examined residual gastric fluid volume and acidity in 245 children. The mean fasting time for clear fluids was 6.9 h (1 h 40 min to 18 h 35 min). The mean pH of the residual gastric fluid was 1.5 (SD 0.9). A reverse correlation (r = −0.236, *p* = 0.01) between residual gastric volume and pH indicates that a lower pH was correlated with larger volumes [31]. Another prospective clinical trial comparing gastric pH and residual volume after one vs. two hours of preoperative clear fluid fasting in 131 children (ASA I or II) aged 1.01 to 16.23 years showed that one hour of clear fluid fasting does not alter gastric pH or residual volume significantly compared with fasting for two hours [30] The mean gastric pH in the one-hour fasting group was 1.44 (SD 0.26) [30] The mean pH of the residual gastric fluid in our study was higher, at 2.14 (SD 1.23), than the previously mentioned studies and did not correlate with age, weight, or time from the end of contrast [5]. We did not evaluate the effect of patient’s medications on gastric pH or gastric fluid volume, as it was beyond the scope of this study.

Although increased gastric contents theoretically increase the risk of aspiration pneumonia, no known gastric fluid volume places a particular patient at clinically relevant risk or eliminates all the risk [36]. Gastric fluid volume has been used as a surrogate marker for pulmonary aspiration risk in studies evaluating fasting protocol safety [21,37,38]. However, the minimum gastric aspirate volume needed to cause complications from pulmonary aspiration remains to be defined. A 1.5 mL/Kg threshold was recently suggested as an appropriate maximum gastric volume, while maintaining low aspiration risk, supplanting the previously accepted recommendation of 0.4–0.8 mL/ Kg [13]. In our study, there was no evidence of pulmonary aspiration in any patient, and only three cases of vomiting occurred in our study, despite having significant residual gastric fluid volume (>1.5 mL/Kg) in 44% of our patients. The data sample in our study is small relative to the reported incidence of aspiration in the literature. Therefore, we cannot make any firm recommendation on the safety of a technique for anesthesia/sedation for patients who receive large amounts of clear fluid one hour prior to anesthesia. The low incidence of aspiration pneumonia with sedation and anesthesia might be because the stomach is a distensible organ that accommodates large amounts of fluids before the resting intragastric pressure rises [39]. Gastric pressure must exceed the barrier pressure of the lower esophageal sphincter for regurgitation to occur. The barrier pressure of the lower esophageal sphincter does not appear to be as easily overcome under general anesthesia as is generally believed [39].

The present study had several limitations. First, this was a small study performed at a single institution, which may cause clinically meaningful differences in treatment effect vs. large multicenter studies. While this trial provides valuable information, it should be used cautiously in clinical decision-making, and future large-scale clinical trials are required to change treatment decisions [40]. Our study lacked a control group—all subjects had comorbid conditions. There may be a difference between the gastric fluid volume of healthy children and children with comorbidities. We assumed that the very diluted oral contrast mixed with standard preoperative clear liquid drinks will mimic the gastric emptying time of standard preoperative clear liquids. All study subjects had to drink a predefined, large amount of clear liquid (>10 mL/Kg), regardless of thirst. We followed standard preoperative NPO instructions, but individual preoperative fluid intake prior to the diluted contrast ingestion and fluid status were not assessed. Finally, we did not evaluate the effect of the anesthesia regimen or medications on gastric fluid volume, as it was beyond the scope of this study. Most patients were induced with propofol, and anesthesia was maintained with sevoflurane. Therefore, our average gastric fluid volume may be affected by the choice of anesthetic [41]. Our study was conducted over a long period of time, during which protocols and procedural changes could have affected the study parameters. However, the majority of our patients (61/70, 87%) were recruited during the first four years of the study period and any unknown protocol/procedural changes during the later study period may not have undue influence on the results.

In conclusion, while studies show that pre-procedural timing and preparation could be considerably facilitated by reducing clear fluid fasting time to one hour in healthy children [1,2,17,18], children with comorbid diseases who receive clear fluids up to one hour before anesthesia can have significant gastric residual volumes. Despite no evidence of increased complications in this study, these data show that many patients will have larger gastric fluid volumes than is recommended. Until results from large multi-center trials are available, children with comorbidities should continue abstaining from drinking unrestricted large amount of clear fluids starting two hours before the scheduled procedure.

## Figures and Tables

**Table 1:** Volume and dilution protocol for enteric contrast material by weight.

Patient’s Weight (Kg)	Omnipaque™ 300 (Oral) (mL)	Diluent (Clear, Non-Carbonated Liquid) Total Vol.-mL (oz)	Weight Range (mL/Kg)
0–4.4	2	90 (3 oz.)	0–20.5
4.5–8.9	4	180 (6 oz.)	20.2–40
9–17.9	6	270 (9 oz.)	15.1–30
18–35.9	8	360 (12 oz.)	10–20
36–45	12	480 (16 oz.)	10.7–13.3
45.1–69	14	600 (20 oz.)	8.7–13.3
>70	18	750 (25 oz.)	10.7

**Table 2:** Descriptive statistics.

Variable	N (%)	Median (Q1, Q3)	Min	Max
Male/Female	38(54):32(46)			
ASA status I/II/III/IV	2/16/47/5 (3/23/67/7)			
Weight (Kg)	70	13.45 (11.12, 15.59)	5.28	30.2
Age (years)	70	2.75 (1.59, 3.87)	0.25	8.09
Time from end of contrast to CT start (min)	69	1.46 (1.3, 1.75)	1.05	2.23
Gastric Fluid from CT scan (mL)	70	13.57 (3.67, 35.45)	0.17	124.68
Gastric Fluid from Aspirate (mL)	69	6 (0, 22)	0	110
Gastric pH	70	2 (1.5, 2)	0	6

**Table 3:** Patients with highest volume/weight (mL/Kg) of gastric volume on the computed tomography image.

Subject	CT Volume (mL)	Weight (Kg)	Calculated Volume Per Weight by CT Image mL/Kg	Gastric Aspirate (mL)	Primary Diagnosis/Comorbidities
GRV056	124.69	15.6	7.99	110	Renal cell carcinoma
GRV002	95.97	12.2	7.87	0	Neuroblastoma
GRV008	108.03	15.57	6.94	0	Dyskeratosis congenita
GRV062	54.13	8.6	6.29	37	Kawasaki disease
GRV004	93.16	17.2	5.42	0	Lymphoproliferative syndrome
GRV038	58.36	13.2	4.42	5	Trisomy 21, AML
GRV065	42.36	9.85	4.3	38	Wiskott–Aldrich syndrome
GRV061	87	20.9	4.16	90	Mesentric adenitis, asthma, chronic abdominal pain
GRV076	81	19.7	4.11	55	HLH
GRV036	35.90	9.8	3.66	0	Metastatic malignant melanoma
GRV064	36.89	10.6	3.48	38	s/p liver transplant, GERD, neutropenia with fever
GRV010	52.01	15.3	3.40	0	Nephrotic syndrome, ESRD, hypertension
GRV049	69.72	20.9	3.34	19	Esophagitis, duodenitis
GRV023	42.51	12.78	3.34	20	Strep pneumonia, bacteremia
GRV026	34.13	11.4	2.99	22	HCC s/p liver transplant
GRV066	24.65	8.4	2.93	13	Renal cyst, GERD
GRV007	30.27	11.6	2.61	28	AML, Seizures
GRV024	24.14	10.1	2.39	7	Pulmonary myofibroblastic tumor
GRV045	30.20	12.7	2.38	3	s/p liver transplant
GRV001	38.52	16.4	2.35	0	Diamond–Blackfan anemia

CT—computed tomography, AML—acute myeloid leukemia, GERD—gastroesophageal reflux disease, HLH—hemophagocytic lymphohistiocytosis, ESRD—end-stage renal disease, HCC—hepatocellular carcinoma.

## Data Availability

The original contributions presented in the study are included in the article; further inquiries can be directed to the corresponding author/s.

## References

[1] Dobson G., Filteau L., Fuda G., McIntyre I., Milne A.D., Milkovich R. (2022). Guidelines to the Practice of Anesthesia—Revised Edition 2022. Can. J. Anaesth..

[2] Frykholm P., Disma N., Andersson H., Beck C., Bouvet L., Cercueil E., Afshari A. (2022). Pre-operative fasting in children: A guideline from the European Society of Anaesthesiology and Intensive Care. Eur. J. Anaesthesiol..

[3] Joshi G.P., Abdelmalak B.B., Weigel W.A., Harbell M.W., Kuo C.I., Soriano S.G., Stricker P.A., Tipton T., Grant M.D., Marbella A.M. (2023). 2023 American Society of Anesthesiologists Practice Guidelines for Preoperative Fasting: Carbohydrate-containing Clear Liquids with or without Protein, Chewing Gum, and Pediatric Fasting Duration—A Modular Update of the 2017 American Society of Anesthesiologists Practice Guidelines for Preoperative Fasting. Anesthesiology.

[4] Keeter S., Benator R.M., Weinberg S.M., Hartenberg M.A. (1990). Sedation in pediatric CT: National survey of current practice. Radiology.

[5] Berger-Achituv S., Zissin R., Shenkman Z., Gutermacher M., Erez I. (2010). Gastric Emptying Time of Oral Contrast Material in Children and Adolescents Undergoing Abdominal Computed Tomography. J. Pediatr. Gastroenterol. Nutr..

[6] Mahmoud M., McAuliffe J., Kim H.Y., Mishra P., Salisbury S., Schnell B., Donnelly L.F. (2010). Oral contrast for abdominal computed tomography in children: The effects on gastric fluid volume. Anesth. Analg..

[7] Kim S.K. (1968). Small intestine transit time in the normal small bowel study. Am. J. Roentgenol. Radium. Ther. Nucl. Med..

[8] Kaufman R., Kransdorf M., Jelinek J., Moser R., Utz J., Brower A., Hudson T., Berrey B., Reeder J., Matz S. (1989). Technical aspects of abdominal CT in infants and children. AJR Am. J. Roentgenol..

[9] Okada Y., Toyama H., Kamata K., Yamauchi M. (2020). A clinical study comparing ultrasound-measured pyloric antrum cross-sectional area to computed tomography-measured gastric content volume to detect high-risk stomach in supine patients undergoing emergency abdominal surgery. J. Clin. Monit. Comput..

[10] Breiman R., Beck J., Korobkin M., Glenny R., Akwari O., Heaston D., Moore A., Ram P., Breiman J.B.R., Yetter E.M. (1982). Volume determinations using computed tomography. Am. J. Roentgenol..

[11] Nagpal P., Prakash A., Pradhan G., Vidholia A., Nagpal N., Saboo S.S., Khandelwal A. (2017). MDCT imaging of the stomach: Advances and applications. Br. J. Radiol..

[12] Mazonakis M., Damilakis J. (2016). Computed tomography: What and how does it measure?. Eur. J. Radiol..

[13] Van de Putte P., Perlas A. (2018). The link between gastric volume and aspiration risk. In search of the Holy Grail?. Anaesthesia.

[14] Harter R.L., Kelly W.B., Kramer M.G., Perez C.E., Dzwonczyk R.R. (1998). A comparison of the volume and pH of gastric contents of obese and lean surgical patients. Anesth. Analg..

[15] Perlas A., Davis L., Khan M., Mitsakakis N., Chan V.W. (2011). Gastric sonography in the fasted surgical patient: A prospective descriptive study. Anesth. Analg..

[16] Van de Putte P., Vernieuwe L., Jerjir A., Verschueren L., Tacken M., Perlas A. (2017). When fasted is not empty: A retrospective cohort study of gastric content in fasted surgical patients. Br. J. Anaesth..

[17] Kharazmi S.A., Kamat P.P., Simoneaux S.F., Simon H.K. (2013). Violating Traditional NPO Guidelines with PO Contrast before Sedation for Computed Tomography. Pediatr. Emerg. Care.

[18] Ziegler M.A., Fricke B.L., Donnelly L.F. (2003). Is Administration of Enteric Contrast Material Safe Before Abdominal CT in Children Who Require Sedation? Experience with Chloral Hydrate and Pentobarbital. Am. J. Roentgenol..

[19] (2017). Practice Guidelines for Preoperative Fasting and the Use of Pharmacologic Agents to Reduce the Risk of Pulmonary Aspiration: Application to Healthy Patients Undergoing Elective Procedures: An Updated Report by the American Society of Anesthesiologists Task Force on Preoperative Fasting and the Use of Pharmacologic Agents to Reduce the Risk of Pulmonary Aspiration*. Anesthesiology.

[20] Vandenbroucke J.P., von Elm E., Altman D.G., Gøtzsche P.C., Mulrow C.D., Pocock S.J. (2007). Strengthening the Reporting of Ob-servational Studies in Epidemiology (STROBE): Explanation and elaboration. Ann. Intern. Med..

[21] Cook-Sather S.D., Liacouras C.A., Previte J.P., Markakis D.A., Schreiner M.S. (1997). Gastric fluid measurement by blind aspiration in paediatric patients: A gastroscopic evaluation. Can. J. Anaesth..

[22] Wittgrove C., Birisci E., Kantor J., Dalabih A. (2017). Gastric volume and its relationship to underlying pathology or acid-suppressing medication. Anesth. Essays Res..

[23] Spencer A.O., Walker A.M., Yeung A.K., Lardner D.R., Yee K., Mulvey J.M., Perlas A. (2015). Ultrasound assessment of gastric volume in the fasted pediatric patient undergoing upper gastrointestinal endoscopy: Development of a predictive model using endoscopically suctioned volumes. Paediatr. Anaesth..

[24] Schwartz D.A., Connelly N.R., Theroux C.A., Gibson C.S., Ostrom D.N., Dunn S.M., Angelides A.G. (1998). Gastric contents in children presenting for upper endoscopy. Anesth. Analg..

[25] Ingebo K.R., Rayhorn N.J., Hecht R.M., Shelton M.T., Silber G.H., Shub M.D. (1997). Sedation in children: Adequacy of two-hour fasting. J. Pediatr..

[26] R Core Team (2017). R: A Language and Environment for Statistical Computing.

[27] Schmitz A., Kellenberger C.J., Lochbuehler N., Fruehauf M., Klaghofer R., Fruehauf H., Weiss M. (2012). Effect of different quantities of a sugared clear fluid on gastric emptying and residual volume in children: A crossover study using magnetic resonance imaging. Br. J. Anaesth..

[28] Sarhan K.A., Hasaneen H., Hasanin A., Mohammed H., Saleh R., Kamel A. (2023). Ultrasound Assessment of Gastric Fluid Volume in Children Scheduled for Elective Surgery After Clear Fluid Fasting for 1 Versus 2 Hours: A Randomized Controlled Trial. Obstet. Anesthesia Dig..

[29] Beck C.E., Chandrakumar T., Sümpelmann R., Nickel K., Keil O., Heiderich S., Boethig D., Witt L., Dennhardt N. (2020). Ultrasound assessment of gastric emptying time after intake of clear fluids in children scheduled for general anesthesia—A prospective observational study. Pediatr. Anesthesia.

[30] Schmidt A.R., Buehler P., Seglias L., Stark T., Brotschi B., Renner T., Sabandal C., Klaghofer R., Weiss M., Schmitz A. (2015). Gastric pH and residual volume after 1 and 2 h fasting time for clear fluids in children. Br. J. Anaesth..

[31] Aschkenasy G., Leder O., Pardes R., Nir E.A., Shteyer E., Orlanski-Meyer E., Gozal Y. (2023). Preoperative clear fluid fasting and endoscopy-measured gastric fluid volume in children. Paediatr. Anaesth..

[32] Cook-Sather S.D., Gallagher P.R., Kruge L.E., Beus J.M., Ciampa B.P., Welch K.C., Schreiner M.S. (2009). Overweight/obesity and gastric fluid characteristics in pediatric day surgery: Implications for fasting guidelines and pulmonary aspiration risk. Anesth. Analg..

[33] Cook-Sather S.D., Tulloch H.V., Liacouras C.A., Schreiner M.S. (1997). Gastric fluid volume in infants for pyloromyotomy. Can. J. Anaesth..

[34] Søreide E., Søreide J., Holst-Larsen M.H., Steen P.A. (1993). Studies of gastric content: Comparison of two methods. Br. J. Anaesth..

[35] Taylor W.J., Champion M., Barry A.W., Hurtig J.B. (1989). Measuring gastric contents during general anaesthesia: Evaluation of blind gastric aspiration. Can. J. Anaesth..

[36] Splinter W.M., Schreiner M.S. (1999). Preoperative fasting in children. Anesth. Analg..

[37] Splinter W.M., Stewart J.A., Muir J.G. (1990). Large volumes of apple juice preoperatively do not affect gastric pH and volume in children. Can. J. Anaesth..

[38] Schreiner M.S., Triebwasser A., Keon T.P. (1990). Ingestion of Liquids Compared with Preoperative Fasting in Pediatric Outpatients. Anesthesiology.

[39] Jones M.J., Mitchell R.W., Hindocha N. (1989). Effect of Increased Intra-Abdominal Pressure during Laparoscopy on the Lower Esophageal Sphincter. Anesth. Analg..

[40] Unverzagt S., Prondzinsky R., Peinemann F. (2013). Single-center trials tend to provide larger treatment effects than multicenter trials: A systematic review. J. Clin. Epidemiol..

[41] Kang J.G., Kim J.K., Jeong H.S., Jung S.C., Ko M.H., Park S.H., Dal Lee B. (2008). A prospective, randomized comparison of the effects of inhaled sevoflurane anesthesia and propofol/remifentanil intravenous anesthesia on salivary excretion during laryngeal microsurgery. Anesth. Analg..

